# Older age and multi-joint external fixator are two risk factors of complications in ulnar lengthening in children with hereditary multiple exostosis

**DOI:** 10.1186/s13018-020-02080-z

**Published:** 2020-11-23

**Authors:** Chao Zheng, Huanli Han, Yujiang Cao

**Affiliations:** 1 Department of Orthopaedics, Children’s Hospital of Chongqing Medical University, 136 Zhongshan 2nd Rd., Chongqing, 400014 People’s Republic of China; 2 Ministry of Education Key Laboratory of Child Development and Disorders, National Clinical Research Center for Child Health and Disorders, China International Science and Technology Cooperation Base of Child Development and Critical Disorders, Chongqing Key Laboratory of Pediatrics, Chongqing Engineering Research Center of Stem Cell Therapy, Children’s Hospital of Chongqing Medical University, Chongqing, People’s Republic of China; 3 Department of Pediatric Hepatobiliary Surgery, Children’s Hospital of Chongqing Medical University, Chongqing, People’s Republic of China

**Keywords:** Hereditary multiple exostosis, Ulna lengthening, Unilateral external fixation, Forearm deformity, Surgical procedure

## Abstract

**Objectives:**

Hereditary multiple exostosis (HME) often involves forearm deformities. The aim of this study was to present the clinical results of 37 children who underwent ulnar lengthening with two different types of unilateral external fixators and to investigate the risk factors of complications.

**Methods:**

We evaluated 37 children with forearm deformities caused by HME treated in our hospital from January 2008 to July 2019. The surgical procedures included resection of exostosis, osteotomy of the ulna, and gradual lengthening of the ulna with a unilateral external fixator. According to the type of fixator they received, the children were divided into two groups: group A received monorail fixators and group B received multi-joint fixators. Radiographic and functional parameters were assessed. Complications were recorded.

**Results:**

All patients were followed-up for an average of 4.6 years (3.0 to 6.5). In both group A and group B, the ulna shortening (US), radial articular angle (RAA), carpal slip (CS), elbow flexion, forearm pronation, supination, and Mayo Elbow Performance Score (MEPS) values improved significantly from preoperatively to postoperatively (*p* < 0.05). However, the ulnar deviation was observed in 4 cases in group B and no cases in group A. According to logistic regression, the difference was only related to age (*p* < 0.05) and the type of external fixator (*p* < 0.05).

**Conclusions:**

Ulnar lengthening with unilateral external fixation is a safe and effective procedure for the treatment of HME. Regarding complications, deviation of the ulna axis was more likely to occur in older children with multi-joint external fixators.

## Introduction

Hereditary multiple exostosis (HME) is a type of autosomal dominant inheritance-induced skeletal dysplasia, with an incidence of approximately 1/50,000 [1]. Approximately 30–60% of cases involve the forearm, affecting the longitudinal growth of the metaphysis and resulting in slow or stagnant growth of the ulna or radius. Deformities such as ulnar shortening, radius curvature, wrist ulnar deviation, and radial head dislocation gradually appear with age [2].

Most forearm deformities in patients with HME are caused by ulna shortening [2–4]. The distal ulna is affected by exostoses, which lead to dysplasia and shortening. Moreover, it tethers the ulnar side of the distal radius, hinders the growth of the distal radius, increases the RAA and CS, and weakens the support provided by the ulna to the wrist joint. On the other hand, the pressure of the radius increases, and with increasing age, the radius bends gradually, resulting in dislocation of the radial head [5]. Therefore, treatments of forearm deformity caused by HME should primarily involve the early correction of ulnar shortening.

The optimal timing and surgical treatment for forearm deformity caused by HME remain controversial [6–8]. The most common treatment is the proximal ulnar osteotomy. Different external fixators are used to gradually lengthen the ulna and correct forearm deformities [7, 9–11]. In particular, the unilateral external fixation is a reliable and effective method with advantages such as being simple to use in operations, leading to minimal surgical trauma, being easy to maintain post-operatively and yielding significant lengthening of the ulna; thus, it has been widely used [12, 13]. However, due to the curvature of the deformity of the ulna itself, the end of the ulna osteotomy region may deviate from the axis during the lengthening process. Considering the age and nutritional status of the patients, reports about various complications, such as the delayed union of the osteotomy end and malunion, are common [14, 15]. Launay et al. [16] inserted an axial Kirschner wire into the ulna to avoid axis deviation and guide the ulna during lengthening. Nevertheless, the relationship between the types of external fixators and complications remains unclear.

Thirty-seven children with forearm deformities caused by HME were enrolled in this study, and they underwent the surgical procedure of ulnar lengthening with two different types of single-arm external fixators. The aim of this study was to retrospectively analyze and present the clinical results and compare the two types of external fixators in terms of the complications that occur postoperatively.

## Methods

### Patients

**Fig. 1:**
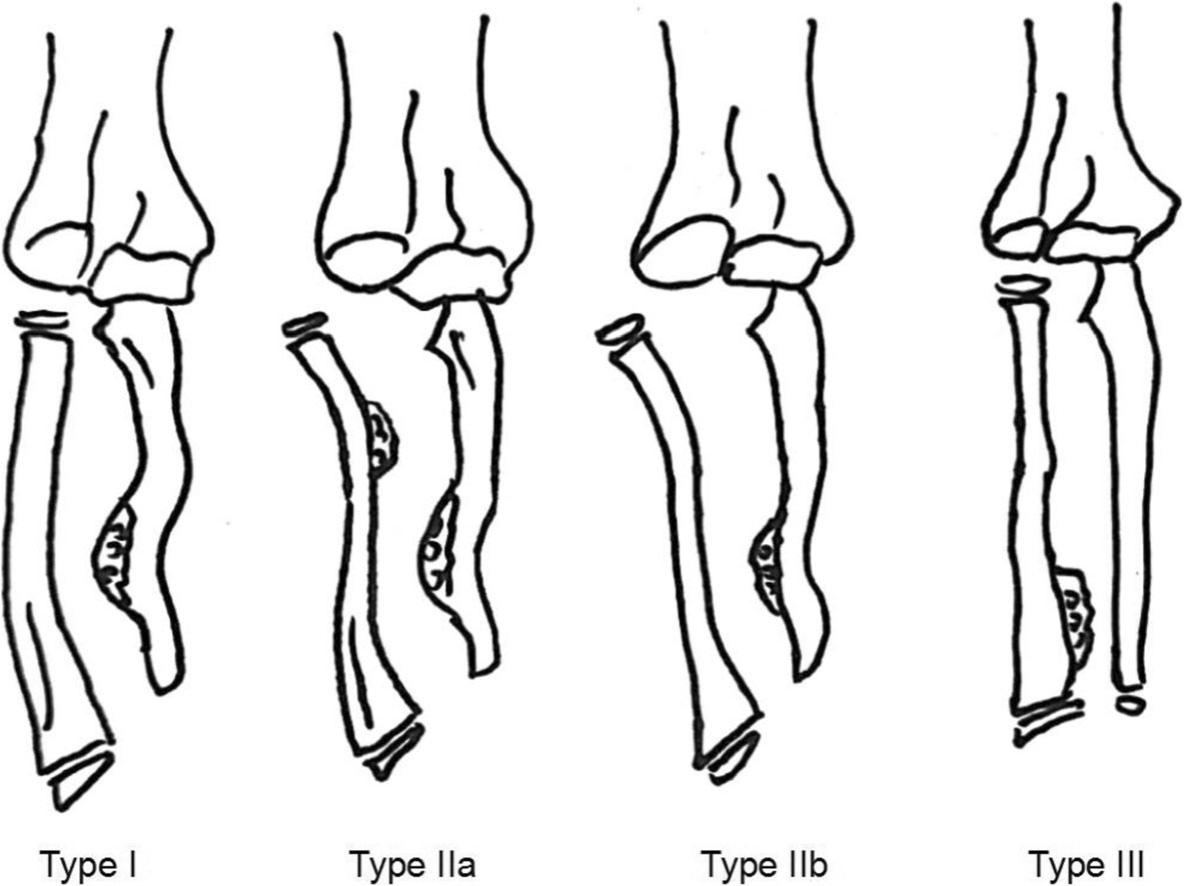
Schematic drawing of the Masada classification for forearm deformity in patients with multiple osteochondromas. Type 1: the main osteochondroma formation is in the distal portion of the ulna, but the radial head is not dislocated. Type IIA: the radial head is dislocated because of an osteochondroma at the proximal metaphysic of the radius. Type IIB: in addition to ulnar shortening the radial head is dislocated. Type III: the main osteochondroma formation is in the metaphysic of the distal radius, and there is relative shortening of the radius. MHE multiple hereditary exostosis

Thirty-seven patients (26 males and 11 females) with forearm deformities caused by HME were treated in our hospital from January 2008 to July 2019. All of the deformities were unilateral. The age at operation ranged from 4.5 to 12.5 years (average 7.4 years). The forearm deformities were classified according to the Masada Classification system [2, 17], which is based on the morphological characteristics of the deformity on plain radiographs (Fig. 1). According to the type of single-arm external fixators applied in surgery, the 37 patients were divided into two groups: group A received monorail fixators and group B received multi-joint fixators. The patients’ basic information is shown in Table 1.

**Table 1:** Patients’ basic information (*n* = 37)

Subject	Group A	Group B	P*
Number (*n*)	27	10	
Age (year)	7.30 ± 1.80 (4.5–12.0)	8.03 ± 2.42 (5.0–12.5)	0.084
Sex (*n*) male to female	22:5	4:6	0.555
BMI (kg/m^2^)	15.8 ± 1.20 (13.6–18.9)	16.72 ± 1.14 (14.8-18.8)	< 0.05
Type (*n*)	I:6; IIa:11; IIb:10; III:0	I:3; IIa:4; IIb:3; III:0	< 0.05
Side (*n*) left to right	15:12	4:6	–
Type of external fixator	Monorail	Multi-joint	–
Follow-up (year)	4.53 ± 1.42 (3.0–6.5)	4.87 ± 1.33 (3.0-5.8)	0.389
US (mm)	23.15 ± 5.55	23.40 ± 4.84	0.801
RAA (°)	32.30 ± 4.07	31.30 ± 5.36	0.353
CS (%)	70.37 ± 6.70	72.30 ± 4.35	0.408
MEPS	74.07 ± 3.68	74.50 ± 3.69	0.775
Elbow flexion (°)	104.26 ± 4.94	106.50 ± 4.74	0.242
Forearm pronation (°)	71.11 ± 3.76	69.00 ± 3.94	0.180
Forearm supination (°)	83.33 ± 3.67	83.00 ± 4.22	0.749

*By Mann-Whitney *U* test, *p* value < 0.05. Statistically significant

The inclusion criteria for surgery included worsening of the deformity or impairments in daily activities, such as ulnar shortening (US) by at least 15 mm or 8% of the length of the ulna [10], associated radial head dislocation, and dysfunction of the elbow, forearm, or wrist. For dysfunction, we evaluated movements of elbow flexion inferior to 110° and pronation inferior to 70°. Elbow extension and forearm supination were not included in the inclusion criteria because they have not been identified as obvious indicators of dysfunction in the clinical literature. The exclusion criteria were as follows: patients with good appearance and function of the upper limb, traumatic dislocation of the radial head, age of less than 3 years old, malnutrition, or any other condition considered a contraindication for operation and anesthesia.

### Surgical procedure

All surgical procedures were performed by the same senior surgeon. Under full general anesthesia, each patient was placed in the supine position. A tourniquet (180–200 mmHg for a maximum of 60 min) and hand table were used. A longitudinal incision was made according to the location and size of the exostosis. The exostosis, as well as the surrounding periosteum, was bluntly separated and completely exposed. Adjacent vessels, nerves, and epiphyses were protected. Four Schanz pins (typically 80 mm in length with 20 mm in thread length, conical blunt-tipped, and hydroxyapatite-coated) were placed on the ulna in parallel to each other and on the same plane, avoiding the plane of maximum curvature on the back of the ulnar arch. The osteotomy was performed using a sharp drill and a chisel whilst preserving the periosteum. The preferred location for osteotomy was the proximal part of the ulna near the epiphysis, avoiding the middle segment of the ulna and leaving enough space for Schanz pins. Then, a monorail single-arm external fixator (Orthopaedic External Fixator Systems, Via delle Nazioni 9,37012 Bussolengo, Verona, Italy) was applied. For complex deformities, when the Schanz pins cannot be placed on the same plane, a multi-joint fixator was applied. In patients with dislocation of the radial head, no special procedures were performed for the radial head.

### Radiological and clinical evaluation

**Fig. 2:**
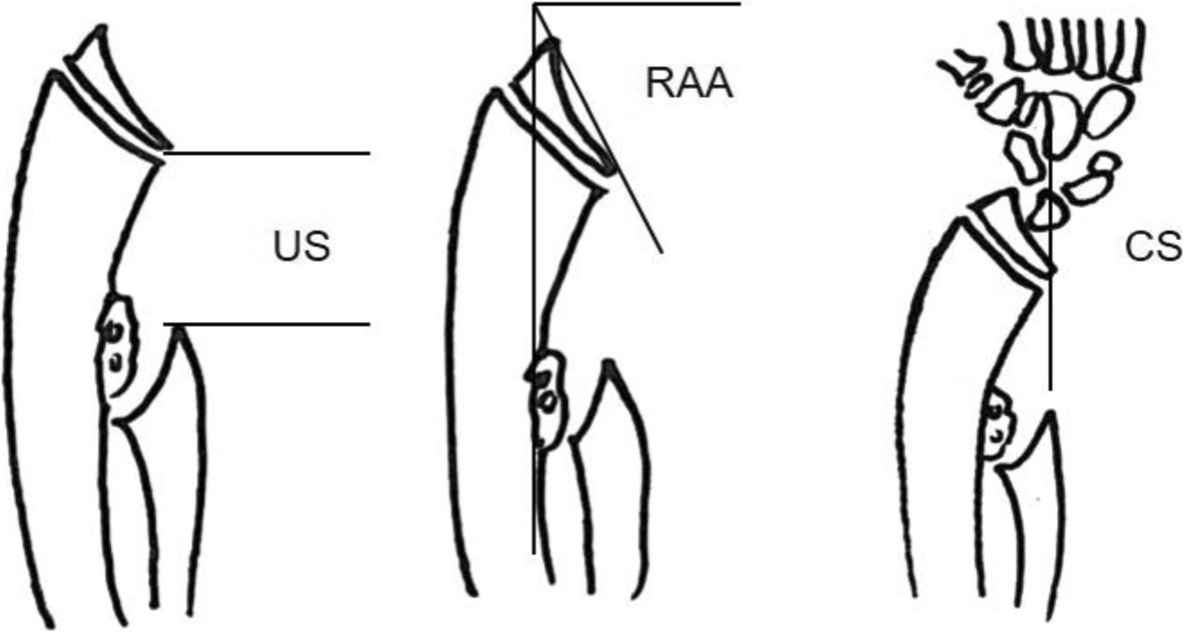
Schematic drawing of US, RAA, and CS. (US, ulna shortening; RAA, radial articular angle; CS, carpal slip [10, 18]). US is measured with the perpendicular line drawn from the distal end of the ulna to the linear axis of the forearm; RAA is the angle between a line drawn along the articular surface of the radius and the other perpendicular to a line that bisects the head of the radius and passes through the radial edge of the distal radial epiphysis; CS was measured as the percentage of the lunate in contact with the radius, as limited by a line drawn from the center of the olecranon through the ulnar edge of the radial epiphysis, which normally bisects the lunate. The CS is abnormal when ulnar displacement of the lunate is more than 50%

According to Fogel et al. [18], the radiological evaluation indicators of forearm deformities caused by HME mainly include ulna shortening (US), the radial articular angle (RAA), and carpal slip (CS) (Fig. 2). The above indexes were recorded preoperatively and at the final follow-up. Deviations in the ulna axis (deviated more than one time of ulna diameter or more than 15°of ulna axis), poorly regenerated bone formation, redislocation of the radial head, the recurrence of an exostosis, and other complications were also recorded. In the clinical evaluations performed preoperatively and at the final follow-up, elbow flexion, and forearm rotation were recorded. The Mayo Elbow Performance Score (MEPS) [19] was used to evaluate elbow function.

### Ulnar lengthening and follow-up

Many methods are available to estimate the lengthening length. To avoid the recurrence of deformity, most scholars suggest that the length of the ulna should be over-lengthened by 4–10 mm more than the length of the contralateral ulna [9, 13, 18]. In this study, the length of the contralateral ulna +4–10 mm was used as the target length. The ulnar was lengthened by 0.25 mm increments done 3–4/day to achieve a distraction rate of 0.75–1 mm/day. The sensation, movement, and circulation of the limbs were closely observed, as well as the increase in the ulna axis and degree of callus at the osteotomy region. Biweekly radiographs were taken for radiographic follow-up. After the predetermined length was reached, lengthening was stopped, and X-ray scans were taken every 1–2 months. When regenerate was healed (the osteotomy line disappeared and the marrow cavity recanalized), the patients were readmitted to the hospital so that the lengthening device could be removed under anesthesia.

### Statistical evaluation

SPSS 26.0 (IBM, Armonk, NY, USA) software and the R programming language (R Core Team 2018. Vienna, Austria) were used to perform all the statistical analyses. For both group A and group B, variables that were measured preoperatively and at the final follow-up, such as the US, RAA, CS, MEPS, and ranges of elbow flexion, forearm pronation, and supination, were assessed by the Wilcoxon test. The improvements in each variable listed above were assessed by Spearman correlation analysis adjusted for variables such as age, sex, type of HME, follow-up time, and increase in the length of the ulna. The differences in complications between the two groups were assessed by two logistic regression models using the R programming language.

## Results

All 37 patients were followed-up. According to the type of external fixator the patients received, they were divided into two groups, and the basic information is shown in Table 1.

**Fig. 3:**
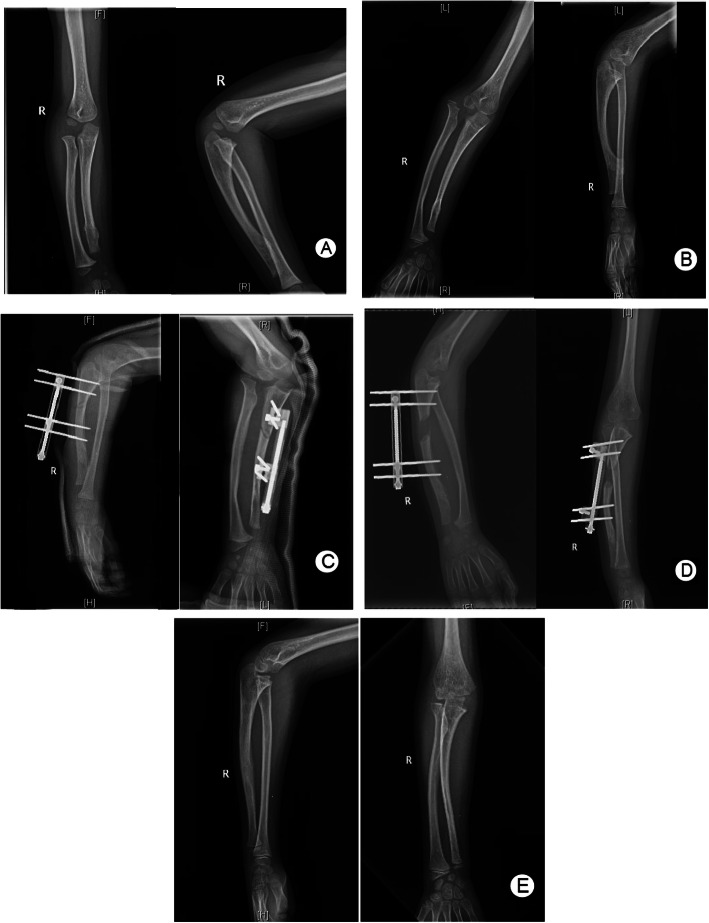
A girl of Masada type IIb forearm deformity: (**a**) 3.5-year-old, X-rays. Forearm deformity was not obvious, with good elbow function. (**b**) 6.5-year-old, preoperative X-rays. Ulnar shortening, ulnar deviation of wrist, radial head dislocation and cubitus varus were obvious, with flexion and pronation disorder of elbow. (**c**) Early postoperative X-rays. Osteochondroma resection and ulna osteotomy were performed, with the Monorail external fixator ready for ulnar lengthening. (**d**) 1 month after operation, 2.5cm were gained. (**e**) 9.5-year-old, X-rays of the latest follow-up. Spontaneous reduction of radial head was observed, and the distal ulna obtained a certain growth potential. The forearm deformity was corrected

In group A, as shown in Table 2, the US, RAA, CS, elbow flexion, forearm pronation, supination, and MEPS values improved from preoperatively to postoperatively. All the changes were found to be significant by the Wilcoxon test (*p* < 0.05). According to Spearman correlation analysis, although changes in RAA and CS were associated with improved length of the ulna (IL) (*p* < 0.05), all the other improvements were not associated with age, sex, the type of HME, follow-up time, or IL (*p >* 0.05). Pin site infections were found in 3 patients, and all patients recovered after oral antibiotics. No other complications were recorded. A typical case is shown in Fig. 3a–e.

**Table 2:** Clinical results of group A

Subject	Preoperative	FFU	Wilcoxon test		*P* value of Spearman correlation analysis				
			*Z*	*P*	Age	Gender	Follow-up	Type	IL^a^
US (mm)	23.15 ± 5.55	0.70 ± 2.49	− 4.546	< 0.05	0.555	0.855	0.239	0.097	Na
RAA (°)	32.30 ± 4.07	22.22 ± 2.28	− 4.550	< 0.05	0.807	0.193	0.184	0.440	< 0.05
CS (%)	70.37 ± 6.70	26.70 ± 3.42	− 4.547	< 0.05	0.863	0.951	0.828	0.558	< 0.05
MEPS	74.07 ± 3.68	94.26 ± 6.46	− 4.541	< 0.05	0.367	0.643	0.080	0.698	0.946
Elbow flexion (°)	104.26 ± 4.94	130.44 ± 5.18	− 4.569	< 0.05	0.263	0.975	0.625	0.460	0.380
Forearm pronation (°)	71.11 ± 3.76	83.52 ± 3.34	− 4.592	< 0.05	0.233	0.403	0.099	0.730	0.308
Forearm supination (°)	83.33 ± 3.67	84.07 ± 3.68	− 2.000	< 0.05	0.384	0.320	0.790	0.670	0.973
Complications (*n*)									
Deviation of ulna axis		0 case							
Poor regenerate bone formation		0 case							
Neurovascular complications		0 case							
Fracture after fixator removal		0 case							
Pin site infection		3 cases							
Redislocation of radial head		0 case							
Recurrence of exostosis		0 case							

*Abbreviations*: *US* ulnar shortening, *RAA* radial articular angle, *CS* carpal slip, *MEPS* Mayo Elbow Performance Score, *FFU* final follow-up, *IL* improved length, *Na* not available, ^a^IL = |US(preoperative)-US(FFU)|*. P* value < 0.05 statistically significant

**Fig. 4:**
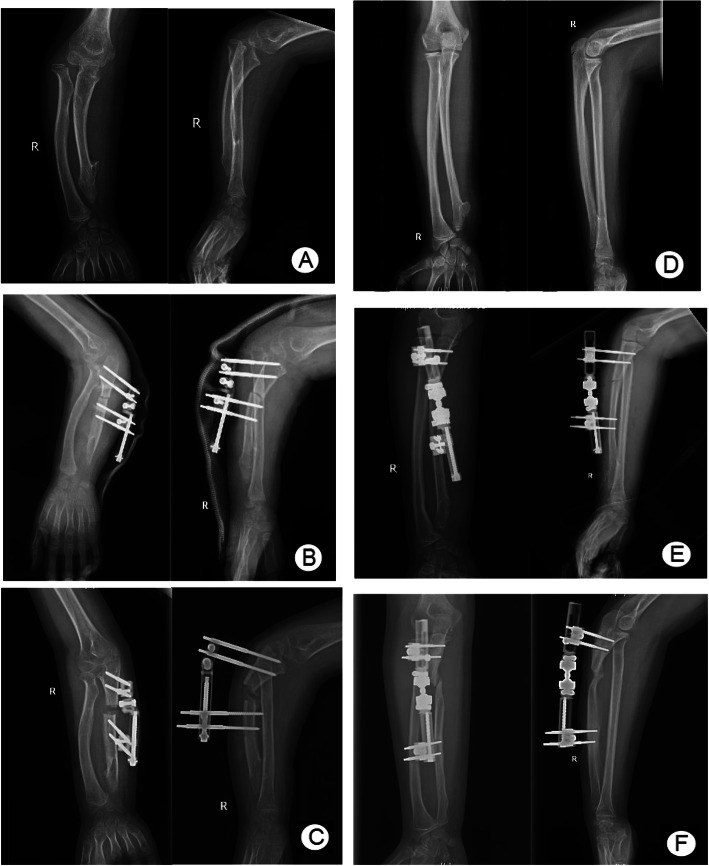
The relationship between the direction of shanz nail and complications. (**a**, **b**) A boy of Masada type IIb underwent surgery at the age of 8.5 with Multi-joint external fixator. Schanz pins were located at the maximum dorsal arch plane of the ulna, and the two groups of pins were not in the same plane (**c**) During one month's lengthening, the ulna gradually developed angulation deformity. (**d**, **e**) A boy of Masada type I received operation at the age of 12.5 with Multi-joint external fixator. The two groups of shanz nails were not in the same plane. (**f**) After 1.5 months' lengthening and 4.5 months of conservative treatment, ulnar healed poorly and showed nonunion

During the process of the gradual extension of the ulna, the reduction of the radial head was satisfying. In group B, as shown in Table 3, the US, RAA, CS, elbow flexion, forearm pronation, supination, and MEPS values improved as well. According to the Wilcoxon test, all the improvements were found to be significant (*p* < 0.05), except for that of supination (*p* > 0.05). The improvements were not associated with age, sex, the type of HME, follow-up time, or IL (*p >* 0.05), according to Spearman correlation analysis. The reduction of the radial head was satisfying in the process of ulna lengthening, and no redislocation was reported. One patient presented with pin site infection and recovered after oral antibiotics. Deviation of the ulna axis and poor bone formation was found in 4 patients, two typical cases are shown in Fig. 4a–f. These cases were resolved by removing the external fixator, inserting a bone block harvested from the autologous iliac crest, and inserting a locking compression plate (LCP) plate.

**Table 3:** Clinical results of group B

Subject	Preoperative	FFU	Wilcoxon test		*P* value of Spearman correlation analysis				
			*Z*	*P*	Age	Gender	Follow-up	Type	IL^a^
US (mm)	23.40 ± 4.84	0.70 ± 3.09	− 2.805	< 0.05	0.724	0.554	0.475	0.619	Na
RAA (°)	31.30 ± 5.36	21.70 ± 3.40	− 2.805	< 0.05	0.337	0.921	0.772	0.525	0.242
CS (%)	72.30 ± 4.35	28.30 ± 3.43	− 2.807	< 0.05	0.148	0.171	0.148	0.333	0.358
MEPS	74.50 ± 3.69	96.00 ± 3.944	− 2.919	< 0.05	0.080	0.486	0.365	1.000	0.554
Elbow flexion (°)	106.50 ± 4.74	127.00 ± 2.58	− 2.850	< 0.05	0.581	0.076	0.462	0.949	0.361
Forearm pronation (°)	69.00 ± 3.94	84.00 ± 3.16	− 2.836	< 0.05	0.064	0.606	0.868	0.251	0.598
Forearm supination (°)	83.00 ± 4.22	83.50 ± 4.12	− 1.000	0.317	0.122	0.447	0.174	0.214	0.122
Complications (*n*)									
Deviation of ulna axis		4 case							
Poor regenerate bone formation		4 case							
Neurovascular complications		0 case							
Fracture after fixator removal		0 case							
Pin site infection		1 cases							
Redislocation of radial head		0 case							
Recurrence of exostosis		0 case							

Abbreviations: *US* ulnar shortening, *RAA* radial articular angle, *CS* carpal slip, *MEPS* Mayo Elbow Performance Score, *FFU* final follow-up, *IL* improved length, *Na* not available. ^a^IL = |US(preoperative)-US(FFU)|*. P* value < 0.05 statistically significant

Differences in complications between the two groups were assessed by two logistic regression models using the R programming language (Table 4). For the deviation of the ulna axis, the difference between the two groups was only related to age (*p* < 0.05) and the type of external fixator (*p* < 0.05) but not to sex (*p* > 0.05), the follow-up time (*p* > 0.05), or the type of HME (*p* > 0.05). The positive estimate values suggested that ulna axis deviation was more likely to occur in older children with multi-joint external fixators.

**Table 4:** Ulnar axis deviation occurred between two groups

Subject	Logistic regression model 1^a^			Logistic regression model 2^b^		
	Estimate value	*t*	*P*	Estimate value	*t*	*P*
Age	0.044 ± 0.021	2.110	< 0.05	0.048 ± 0.020	2.376	< 0.05
Gender	0.083 ± 0.099	0.838	0.409	–	–	–
Follow-up	− 0.015 ± 0.031	− 0.483	0.632	–	–	–
TypeIIa	− 0.032 ± 0.106	− 0.305	0.763	–	–	–
TypeIIb	0.179 ± 0.103	1.737	0.093	–	–	–
Type of external fixator	0.415 ± 0.099	4.197	< 0.05	0.360 ± 0.093	3.884	< 0.05

Assignment:Type I, Type IIa, Type IIb: 0,0; 1,0; 0,1; Gender:male:1; female:0; Type of external fixator; Monorail:0; Multijoint: 1

^a^Logistic regression model with multiple variables included

^b^Logical regression model after eliminating irrelevant variables on the basis of model 1

## Discussion

The aim of this study was to present the clinical results of 37 children who underwent ulnar lengthening with two different types of unilateral external fixators and to investigate the risk factors of complications.

In this study, two types of unilateral external fixations were selected: the monorail (group A) and multi-joint fixators (group B). Tables 2 and 3 show that the US, RAA, CS, elbow flexion, forearm pronation, and MEPS values significantly improved in both group A and group B (*p* < 0.05). Only supination in group B did not significantly change, which might be attributed to the small sample size. The appearance and function of the upper limb significantly improved in the two groups, and the effect of unilateral external fixation on ulnar lengthening was obvious.

For complications, no fractures, neurovascular problems, or recurrences of exostoses were observed in the two groups. Pin site infections (5 cases in total) easily recovered. For radial head dislocation in patients with type IIa and type IIb deformities, no special procedures were performed during the operation, all cases self-reduced during ulna lengthening, and no cases of redislocation were found. This result suggests that it may be best not to treat radial head dislocation. The interosseous membrane can transmit the forces leading to lengthening, and the reduction of the radial head can be reached gradually [20].

Remarkably, ulna deviations and poorly regenerated bone formation were more likely to occur in group B than in group A. Differences between the two groups were assessed by two logistic regression models using the R programming language (Table 4), which suggested that older age and multi-joint external fixator are two risk factors.

Older age was one risk factor for ulna deviations and nonunion in this study. The regenerate healed more slowly in older age patients than that in younger ones; therefore, it was easy to be affected by other factors in the process of lengthening and lead to nonunion. It may suggest that surgical intervention should be performed at an earlier age. The optimal timing of surgical intervention is controversial. One view [2, 12, 21, 22] is that early surgery can slow or prevent the progression of deformities, especially the dislocation of the radial head, while for patients with dislocation, early surgery often leads to self-reduction. Another view [14, 23, 24] is that surgery should be postponed until the patient is 10 years old or at the age of epiphyseal closure. We believe that the timing of surgery should be determined on the basis of not only age but also the actual condition of the patients. For patients with an obvious forearm and wrist deformities, a US value larger than 1.5 cm [22], radial head dislocation, enlargement of an exostosis, dysfunction, or chronic pain, the operation should be performed early, which can reduce the incidence of complications. When the radial head has been dislocated for a long time, the morphology of the humerus and radius joint, annular ligament, and other soft tissue structures may change, the failure rate of surgical reduction is high, and the function of the forearm may be poor. As shown in Fig. 3a–e, at the age of 3.5 years, a girl had good forearm appearance and function, without dislocation of the radial head. At the age of 6.5 years, the radial head was dislocated, the upper limb force line was obviously skewed, elbow flexion and forearm rotation were limited, and the operation was performed. At the 3-year follow-up, the reduction of the radial head was satisfying, and the ulnar shortening deformity was corrected.

Another risk factor for ulna deviations and nonunion was the use of multi-joint external fixator. In some cases of complex deformities, the pins of the fixator were not orthogonal and not suitable for mono-rail fixator, so we used a multi-joint fixator to adapt to the divergence angle of pins. However, 4 out of 10 cases who were lengthened by the multi-joint fixator resulted in poor bone formation. It suggested that instability may be a potential defect of a multi-joint fixator. Although multi-joint fixations were stable and reliable in the surgical treatment of fractures, there was still instability throughout the process of ulnar lengthening, whether human factors or instrumental factors. On the other hand, for complex malformations, when the four pins were not located in the same plane, lateral torque will be generated during the ulna lengthening, resulting in ulna deviation. Therefore, we suggest that for complex deformities, more stable fixators, such as the Ilizarov fixator [7, 9], may be more suitable.

The occurrence of nonunion may also be related to the position of the osteotomy and direction of the nail. It has been reported that [15] the diameter of the osteotomy site is negatively related to the time of bone healing. Some scholars [13] have suggested that the osteotomy point should be located at the maximum bending point, and the distance between the osteotomy point and the distal end of the ulna should be larger than 42% of the total length of the ulna. We suggest that the proximal part is selected, avoiding the maximum curvature as much as possible. Four Schanz pins were placed in the same plane in parallel to each other, fixed with monorail fixators.

A patient with type IIb deformity is shown in Fig. 4a–c. The two groups of Schanz pins were located at the maximum bending plane of the ulna arch, and the nails were not positioned in parallel to each other or in the same plane. During the lengthening process, the joint of the external fixator became loose, and the dorsal angle of the ulnar arch gradually increased. Another patient with a type I deformity is shown in Fig. 4d–f. Although the Schanz pins avoided the maximum bending plane of the ulna arch, they were positioned in parallel to each other; however, they were not located in the same plane, so the force line was skewed, and the ends of the osteotomy region were separated during the lengthening process, leading to nonunion.

Whether exostoses should be removed is still debated. Akita [14] et al. found that exostosis resection can significantly improve the rotational function of the forearm but that it affects the US, RRA, and CS values very little. We believe that the resection of exostoses can open the epiphysis of the distal ulna, enable the ulna to obtain a certain growth potential, reduce the effect of local tissue on the radius, and correct deformities of the wrist. In this study, 37 patients underwent exostosis resection. The function of the forearm significantly improved.

Our study presents some limitations. This study is a retrospective study, with a small sample size and a short follow-up time. The clinical efficacy and complications remain to be further verified by a long-term prospective randomized controlled study.

## Conclusions

Ulnar lengthening with unilateral external fixation is a safe and effective procedure for the treatment of HME. Older age and multi-joint external fixator are two risk factors of complications. Monorail fixators are more reliable than multi-joint fixators.

## Data Availability

The datasets analyzed in the study are available from the corresponding author on reasonable request.

## Abbreviations

HME: Hereditary multiple exostosis
US: Ulna shortening
RAA: Radial articular angle
CS: Carpal slip
FFU: Final follow-up
IL: Improved length

## References

[1] Schmale GA, Conrad EU, Raskind WH (1994). The natural history of hereditary multiple exostoses. Jbjs.

[2] Masada K, Tsuyuguchi Y, Kawai H (1989). Operations for forearm deformity caused by multiple osteochondromas. J Bone Joint Surg.

[3] Jo AR, Jung ST, Kim MS (2017). An evaluation of forearm deformities in hereditary multiple exostoses: factors associated with radial head dislocation and comprehensive classification. J Hand Surg.

[4] Stieber JR, Dormans JP (2005). Manifestations of hereditary multiple exostoses. J Am Acad Orthop Surg.

[5] Gottschalk HP, Kanauchi Y, Bednar MS (2012). Effect of osteochondroma location on forearm deformity in patients with multiple hereditary osteochondromatosis. J Hand Surg.

[6] Noonan KJ, Levenda A, Snead J (2002). Evaluation of the forearm in untreated adult subjects with multiple hereditary osteochondromatosis. Jbjs.

[7] Hill RA, Ibrahim T, Mann HA (2011). Forearm lengthening by distraction osteogenesis in children: a report of 22 cases. J Bone Joint Surg Br Vol.

[8] Beutel BG, Klifto CS, Chu A (2014). Timing of forearm deformity correction in a child with multiple hereditary exostosis. Am J Orthop.

[9] Ahmed AARY. Gradual ulnar lengthening by an Ilizarov ring fixator for correction of Masada IIb forearm deformity without tumor excision in hereditary multiple exostosis: preliminary results. J Pediatr Orthop B. 2019;28(1):67–72. 10.1097/BPB.0000000000000514 29995654

[10] D’Ambrosi R, Barbato A, Caldarini C (2016). Gradual ulnar lengthening in children with multiple exostoses and radial head dislocation: results at skeletal maturity. J Child Orthop.

[11] Farr S, Mindler G, Ganger R (2016). Bone lengthening in the pediatric upper extremity. J Bone Joint Surg.

[12] Refsland S, Kozin SH, Zlotolow DA (2016). Ulnar distraction osteogenesis in the treatment of forearm deformities in children with multiple hereditary exostoses. J Hand Surg.

[13] Iba K, Hanaka M, Ozasa Y, et al. Treatment of forearm deformity with radial head dislocation because of multiple osteochondromas. J Pediatr Orthop B. 2018;27(4):315–21. 10.1097/BPB.0000000000000453 28306622

[14] Akita S, Murase T, Yonenobu K (2007). Long-term results of surgery for forearm deformities in patients with multiple cartilaginous exostoses. JBJS.

[15] Li Y, Han B, Tang J, et al. Identification of risk factors affecting bone formation in gradual ulnar lengthening in children with hereditary multiple exostoses: a retrospective study. Medicine (Baltimore). 2019;98(5):e14280. 10.1097/MD.0000000000014280 PMC6380801 30702592

[16] Launay F, Jouve JL, Viehweger E (2004). Progressive forearm lengthening with an intramedullary guidewire in children. J Pediatr Orthop.

[17] Aita M, Rodrigues F, Bernardo R (2018). Ulnar lengthening/reconstruction of interosseous membrane in treatment of osteochondroma. J Wrist Surg.

[18] Fogel G. Management of deformities of the forearm in multiple hereditary osteochondromas. J Bone Joint Surg Am. 1984;66(5):670–80. 6725315

[19] Cusick MC, Bonnaig NS, Azar FM (2014). Accuracy and reliability of the Mayo Elbow Performance Score. J Hand Surg.

[20] Mcginley JC, Kozin SH (2001). Interosseous membrane anatomy and functional mechanics[J]. Clin Orthop Relat Res.

[21] Litzelmann E, Mazda K, Jehanno P (2012). Forearm deformities in hereditary multiple exostosis: clinical and functional results at maturity. J Pediatr Orthop.

[22] Pritchett JW (1986). Lengthening the ulna in patients with hereditary multiple exostoses. J Bone Joint Surg Br Vol.

[23] Ham J, Flipsen M, Koolen M (2016). Multiple osteochondromas (MO) in the forearm: a 12-year single-centre experience. Strategies Trauma Limb Reconstr.

[24] Arms DM, Strecker WB, Manske PR (1997). Management of forearm deformity in multiple hereditary osteochondromatosis. J Pediatr Orthop.

